# Reflex Effects of Dental Irritation

**Published:** 1889-11

**Authors:** Albert P. Brubaker

**Affiliations:** Philadelphia, Pa.


### International Dental Journal.

Vol. X. November, 1889. No. 11.

### Original Communications.[1]

### REFLEX EFFECTS OF DENTAL IRRITATION.[2]

BY ALBERT P. BRUBAKER, M.D., D.D.S., PHILADELPHIA, PA.

It is a truth no longer needing confirmation that the teeth, through the mediation of the trifacial nerve, are intimately associated with many, if indeed not all, of the organs of the body; that they are no longer to be regarded as inert structures lying on the outskirts of the physiological domain, and playing no important *rôle* in its activities. On the contrary, recent studies in physiology, and more particularly in pathology, have demonstrated the close interdependence of the teeth and other organs of the body, near and remote; and that, while in a normal condition, the teeth exert an influence upon the nutrition of the organs which is silent and inappreciable, yet, in a pathological condition, they often induce serious functional and organic lesions, the true cause of which is often wholly ignored.

To the experienced practitioners of dental medicine pathological states of organs, which have for their cause dental irritation, have presented themselves so frequently that reference to them may indeed seem trite. To the younger men of the profession, as

[1] The editor and publishers are not responsible for the views of authors of papers published in this department, nor for any claim to novelty, or otherwise, that may be made by them. No papers will be received for this department that have appeared in any other journal published in this country. The journal is issued promptly on the 15th of the month.

[2] Read at the twenty-first annual meeting of the Pennsylvania State Dental Society, held at Cresson, Pa., July 31, 1889.

well as those who are about to engage in dental work, it may not be amiss to summarize what is known of the far-reaching influences of the disorders of the teeth, and to point out the necessity of studying the organism in its entirety, if they hope to obtain broad and general views of dental medicine.

I am impelled to allude to this last point, inasmuch as the observation is frequently made by students entering upon the duties of college life that systematic study of anatomy and physiology is largely unnecessary, and that time which might be employed more advantageously is practically wasted, much to the detriment of the student. The majority of students are possessed with the idea that all the anatomical and physiological knowledge necessary for the practice of their profession is bound up in that of the fifth nerve. But of what use even an exhaustive knowledge of this important nerve, unless it be considered in its anatomical and physiological relations to all other nerves, nerve-centres, and correlated organs?

The design of this paper is, first, to collate briefly the several hundred cases of disease which have been reported from time to time, and which had for their origin pathological conditions of the teeth and associated parts; and, second, to try to show from what is known of the connections of the fifth nerve with the nerve-centres at the base of the brain how these conditions may be brought about. Owing to the inconsequential nature of the nervous response to morbid irritation of the dental and alveolar nerves, no anatomical or scientific order will be attempted in arranging these cases, but I shall consider them in the order of their removal from the inciting causes, beginning with peripheral organs, such as the eye and ear, muscles, etc.; thence to the disorders of nerves and subordinate nerve-centres; and, lastly, to the diseases of the higher co-ordinating centres themselves.

Ocular Diseases.—That ocular disorders may be excited by morbid dental irritation is a fact which has been recognized only in recent times; but within the past few years the concurrent testimony of a large number of investigators point conclusively to this possibility. The causal relation between diseases of the teeth and eye has been shown to exist so frequently that no less an authority than Professor Galezowski, the distinguished French oculist, states that he makes it an invariable rule to first examine the teeth in every case that is brought to his notice. Every structure of the eyeball has been found to bear the brunt of the abnormal condition of the nerve-centres. A study of the cases so far reported will show that the majority are not inflammatory, but rather functional, in character, such as might be expected to result either from over-stimulation or inhibition of nerve-centres. Nevertheless, it can scarcely be doubted that as both vaso-motor and trophic nerve-fibres, whose function it is to regulate the blood-supply and to govern the metabolism of the tissues, also pass to the eye through the ophthalmic division of the fifth nerve, any mal-arrangement of such fibres may give rise to inflammatory conditions.

There have been reported eleven cases of paralysis of ocular muscles, three cases of severe neuralgia of the eyeball, one case of copious lachrymation of one year's standing, twelve cases of inflammation and ulceration of the cornea and sclerotic, twenty-two cases of partial and complete unilateral blindness, all of which had for their direct and exciting cause irritation of the teeth, as was shown by their disappearance upon removal of the irritation.

It has also been shown by Professor Schmidt that the power of accommodation—that power which the eye possesses of focussing the rays of light so as to form distinct images of near objects upon the retina—was impaired in seventy-three out of ninety-two cases troubled with dental caries. As a general rule, the defective accommodation was unilateral, and present upon the side of the dental lesion, though this was not invariable. It is quite evident that an unequal accommodative power would lead to great inconvenience in the performance of accurate work, and to the necessity of wearing glasses prematurely. Most remarkable are those cases of blindness of one eye, which have persisted for a period varying from a few months to twelve years, disappearing in a short time after the extraction of the carious teeth. It is questionable if any other condition is so prolific in the production of this form of functional blindness.

Aural Diseases.—That aural troubles are excited through reflex action from irritation of the dental nerves is also a truth no longer questioned. The fact has become so apparent as to be well recognized by aural surgeons. Thus, out of eighty infants under fourteen months of age examined by Dr. Wreden, of St. Petersburg, more than eighty per cent. had some form of ear trouble. Sexton, in reviewing his records of fifteen hundred cases, says he thinks "perhaps one-third owe their origin or continuance in a greater or less degree to diseases of the teeth." The same writer further says, "That in no case of dead (pulpless) teeth, however carefully treated and filled, can it be ever successfully demonstrated that a slight irritation is not constantly present, although no appreciable irritation may be experienced by the patient." Woakes, who has written an admirable monograph upon ear affections, has accorded the fullest recognition to the dental origin of many ear affections.

Acute and chronic inflammations of the external and middle ear-chambers, both the catarrhal and purulent forms, often leading to complete destruction of the ear-structures, have been shown so often to be due to dental irritation that it is only necessary to allude to it. In addition to the numerous cases of inflammatory diseases that have been reported, at least eight cases of severe and protracted neuralgia of the auditory nerve, which had resisted all means of treatment except removal of the source of irritation.

Leaving out of consideration the half-dozen cases of temporary deafness accompanied by ringing noises in the ear, which had for their origin the same irritation, there is a far more serious form of deafness, first described by Dr. Cooper, of Dublin, which is a concomitant of severe and protracted eruption of the wisdom-tooth. It is a slow, progressive, and intractable form of chronic otitis, for the relief of which neither the extraction of the teeth nor any form of medication has been of any benefit. The irritation excited by the abnormal evolution of the tooth results in an insufficient innervation of the deeper constructures, and causes not a temporary, but permanent alteration in structure. If it could be demonstrated that these two conditions stand in the relation of cause and effect, we would have here another argument in support of the position taken by Professor C. N. Pierce at the last annual meeting, that the extraction of the sixth-year molar may at times be justifiable in order to prevent the development of serious troubles later in life. Especially would this be so in all those cases in which there is an hereditary predisposition to failure of hearing. It would at least eliminate one source of this distressing disorder. Considering the serious consequences that follow individuals deprived of their hearing, the possibility of this condition being caused by wisdom-tooth eruption should engage the attention of all dental surgeons. It can not be doubted that much deafness is a result of unrecognized troubles in early life; and as the condition of deaf-mutism is so often the sequel of ear-troubles, it is highly probable that the great number of these pitiable objects in charitable institutions might be lessened by so simple a matter as attention to the gums during teething.

Where the results of dental irritation are so disastrous as in organs so delicately organized and so complex as the eye and ear, how important it becomes for all dentists to familiarize themselves with their structure, to be awake as to the possibility of dental irritation disordering these structures, as well as the means to avert such disorders! They will then do honor to themselves, to their profession, and at the same time relieve much distress and suffering.

Muscular Disorders.—Under this head will be considered only these instances of active contraction or spasm of voluntary muscles. With the exception of the ocular muscles, the only muscles known to be affected with spasm from dental irritation are the facial group, the masseter, and the sterno-cleido-mastoid. That these muscles should remain in this state of active contraction for an indefinite period without becoming exhausted is somewhat difficult to explain, but it is in all probability due to the fact that the muscular fibres as a whole do not contract simultaneously, but successively, so that while one set is in contraction another set is in repose. This explanation of the condition of these muscles receives some support from what is known of the sphincter muscles, in which this successive contraction of the muscular bands has been shown to exist. It is to be regarded as due to an incessant outflow of nerve-force from the centres kept up by the peripheral irritation.

Ten cases of persistent spasm of the masseter muscles have been recorded, three cases of spasm of facial muscles, and one case of wry neck. Although it is well known that the central origin of the motor nerves innervating these muscles are closely connected by commissural fibres with the origin of the fifth, it is wholly unknown why the peripheral irritation of the trigeminal branches should in one instance be transferred to the facial, and in another to the spinal accessory.

Visceral Disorders.—Neuroses peculiar to the alimentary canal, larynx, heart, and even uterus, arising as a sequence to dental irritation, have from time to time been observed by clinicians. That dental irritation should be reflected to these organs might at first glance seem remarkable, but it is not more so than that mental emotions should result in arrest of the secretion of gastric juice, or that ear-lesions should cause laryngeal cough, or that a blow on the abdomen should paralyze the heart. Turning to clinical medicine for illustrations of visceral disorders, we find that cases of obstinate vomiting, gastric and intestinal irritation, often accompanied by an intermittent fever, laryngeal irritation, palpitation of the heart, and even uterine pains, have been recorded from time to time.

Trophic and Vaso-Motor Disorders.—It is now pretty well established that in addition to the vaso-motor nerves which regulate blood-supply, there are a number of centres and nerves which regulate the metabolism of the tissues. Injury to these nerves or impairment of their centres of origin give rise to more or less serious alteration in structure in various parts of the body. As instances of trophic disorders caused by dental irritation, may be mentioned ulcerations of the surfaces of the cheeks, ulcerations of the cornea, diseases of the skin, of which a number of cases have been reported. Dr. Mulreany, of New York, has been at great pains to establish a relationship between the irritation occurring during the eruption of the first four molar teeth and the appearance at that time of hip-joint disease. There would seem to be some truth in this view, inasmuch as the several cases that he records improved as soon as the irritation was relieved,—that is, as soon as the teeth had erupted.

Owing to want of time, I shall merely allude to the affections of the subordinate nerve-centres. These comprise, first, the various forms of neuralgia, chief of which is that of the fifth nerve, though neuralgia of the brachial and sciatic plexuses is far from being uncommon; second, various forms of paralysis, the most common of which is paralysis of the seventh nerve, or portio dura, leading to complete relaxation of the muscles of the face. At least ten cases of this affection have been recorded. Other paralyses, such as arm and leg paralysis, and even that most unfortunate form, infantile paralysis, have been observed to recover once the irritation of the dental nerves was relieved. Tetanus, four cases of which have thus far been recorded.

The affections of the higher nerve-centres comprise the various forms of headaches, epilepsy, chorea, hysteria, and insanity. Neuralgic headaches, of months' and years' duration, have yielded promptly after removal of impacted and carious teeth, exostosed roots, etc. Numerous are the cases which have been observed by both dentists and physicians. That epilepsy should be excited by dental irritation is at first sight rather remarkable, but it is no more so than that it should arise from any other form of peripheral irritation. Up to the present time some sixteen cases have been reported, all of which entirely recovered after removal of the irritation. Chorea, especially that form limited to the face and shoulders, has been observed in a few instances only, but it is highly probable that a closer examination into the causation of this disease would show that the teeth are often important factors. Hysteria, hystero-epilepsy, and insanity have been caused in not a few instances by carious and ulcerated teeth in highly nervous and excitable patients. From what has been said, it is quite evident that morbid irritations of the teeth are capable of producing extensive pathological conditions in organs near and remote, and that the sphere of the dentist will widen if looked at from this point of view, and will become a valuable ally in the elucidation of many obscure and perplexing problems of clinical medicine.

The explanation of the mechanism and derangement of mechanism by which the pathological states resulting from dental irritation are brought about is a problem much more readily stated than solved. Its solution requires a knowledge of the anatomy and physiology of that complex system of sensory, motor, vaso-motor, and trophic nerve-fibres by which the peripheral organs are harmonized and co-ordinated in their physiological action. The fundamental action, however, is a reflex action, the mechanism of which is quite simple and generally well known. From an irritated point a centripetal influence passes inward to a nerve-centre, from which another impulse travels outward, which excites activity in a muscle, a gland, or other tissue. In the spinal cord and medulla oblongata there are innumerable centres from which nerve-fibres pass to all structures about the head and trunk of the body. These centres are in function, motor, vaso-motor, secretory, trophic, etc., and many of them are in direct anatomical connection with the origin of the fifth nerve. If this or any other sensory nerve be abnormally stimulated at its periphery, the nerve-impulses thus generated travel inward to the centre of origin of that nerve; from thence it may pass in various directions to other centres, according to circumstances of which we know nothing. For example, an impulse reaching the spinal cord or medulla oblongata may be reflected to a motor centre, and the result will be increased contraction of a muscle; or it may be reflected to a blood-vessel, and the result is a variation in the blood-supply, or to a gland, and increased secretion results. In all cases there is an increase in the quantity of nerve-force sent outward to these various structures, with the result of largely increasing their activity. Sometimes, however, the impulse which travels inward produces the very opposite condition of these nerve-centres, produces a condition of inhibition or rest, no nerve-force passing outward. The structures normally innervated by them are paralyzed, and often undergo a change in nutrition. In this way we may explain the forms of paralysis that have been mentioned.

Again, the nerve-force generated at the point of irritation, after travelling inward, may neither be reflected outward, nor produce inhibition of nerve-centres, but it may be stored up in the higher centres until morbid conditions arise, which manifest themselves as headache, epilepsy, insanity, etc.

It is clearly evident that as long as the stimulation of the dental and alveolar nerves is of a normal character, the resulting reflex influences are such as to insure a healthful activity of the organism; but when this irritation becomes painful or morbid, the normal mechanism is deranged and pathological processes incited. Any attempt to trace out specifically the mistaken routes of the nervous currents, the reason of their deroutation, and the rationale of the disordered process is, in the present state of neurology, either impossible or highly unsatisfactory.

To help our minds, we may crudely picture the central nervous system, and particularly the medulla oblongata, as a sort of intricate switch-board of a large telegraph-office, where are focalized myriad wires from all parts of the compass. When in normal action the connections are such that a message from a peripheral point—for example, the dental nerves—is shunted to its proper receiver, transferred to another wire, or sent to higher and other offices. Most of the pathological instances referred to appear to be a result of a disordered condition of the switch-board, the medulla, the result of morbid messages from the periphery. As to any adequate conception or comprehension as to the workings of the medulla in health or disease, we are very much like an individual wholly ignorant of and standing before the switch-board of the unheard-of telegraphic machines.

If what I have said will convince any one of the desirability on the part of dentists of acquiring a wider knowledge of anatomical physiological and pathological knowledge, my object will have been obtained.

